# Evaluation of the Diagnostic Value of Hematologic Parameters and Ratios in SARS-CoV-2 VOC-202012/01 Mutant Population

**DOI:** 10.7759/cureus.28285

**Published:** 2022-08-23

**Authors:** Murat Seyit, Esin Avci, Atakan Yilmaz, Aykut Kemanci, Ahmet Caliskan, Mert Ozen, Alten Oskay, Hulya Aybek, İbrahim Türkcuer

**Affiliations:** 1 Emergency Medicine, Pamukkale University, Denizli, TUR; 2 Medical Biochemistry, Pamukkale University, Denizli, TUR; 3 Emergency, Pamukkale University, Denizli, TUR; 4 Emergency Medicine, Tavşanlı Doc. Dr. Mustafa Kalemli State Hospital, Kütahya, TUR; 5 Medical Microbiology, Pamukkale University, Denizli, TUR; 6 Emergency Department, Pamukkale University, Denizli, TUR

**Keywords:** plr, nlr, platelet, neutrophil, rdw-sd, voc-202012/01, sars-cov2, covid-19

## Abstract

Introduction

In this study, we set out to study possible differences between individuals with and without VOC 202012/01 variant by using less costly complete blood count analytes and quickly analyzing the samples and ratios derived from these analytes. For this purpose, we assessed neutrophil, lymphocyte, platelet, and Red Blood Cell Distribution Width-Standard Deviation (RDW-SD) levels among complete blood count parameters (CBC) (identification and count of red blood cell, neutrophil, eosinophil, basophil, lymphocyte, monocyte, platelet) as well as the neutrophil-lymphocyte ratio (NLR) and platelet-lymphocyte ratio (PLR).

Methods

A retrospective cross-sectional study was performed over the course of two months (from May to June 2021) on 212 patients who presented to the emergency department of a tertiary hospital with Covid-19 symptoms and took SARS-CoV2 PCR and CBC tests. The polymerase chain reaction (PCR)-confirmed SARS-CoV2 positive patients and their hospitalization data were gathered from the public health management system. Their VOC-202012/01 mutation status was also confirmed by this system.

Results

RDW-SD, RDW, NLR, and PLR indexes, as well as C-reactive protein (CRP), and lactate dehydrogenase (LDH) values, were higher in the patients with VOC-202012/01 mutation (p<0.0001) than those without mutation, while hemoglobin and hematocrit counts and ratio, as well as eosinophil and lymphocyte counts, remained lower in the patients with mutation (p<0.0001).

Conclusion

NLR and RLP ratios derived from hematological parameters and models based on these ratios and RDW-SD are cheaper and more widely used. Our study suggests that the hematological analytes, the ratios obtained from these analytes, and the models created through these ratios in patients presenting to the ED with COVID-19-like symptoms and having positive reverse transcription polymerase chain reaction (RT-PCR) test results were significantly different in those with and without the VOC-202012/01 mutation. The bottom line is that they can serve as reliable predictors in the assessment of patients with the VOC-202012/01 mutation.

## Introduction

The novel coronavirus pandemic that broke out in Wuhan, China towards the end of 2019 was named “COVID-19” (coronavirus disease 2019) by the World Health Organization (WHO), and the virus causing it was named “severe acute respiratory syndrome coronavirus 2” (SARS-CoV-2) by the International Committee on Taxonomy of Viruses [1]. SARS-CoV-2, a member of the betacoronavirus subgroup, operates in a fashion remarkably similar to SARS-CoV, primarily targeting the respiratory, hematopoietic, hemostasis, and immune systems.

With 464,809,377 confirmed cases, 6,062,536 confirmed deaths, and 10,925,055,390 vaccine doses administered (as on March 21, 2022), SARS-CoV-2 has developed into a public health emergency of international concern, followed by the emergence of multiple variants [2]. One of these mutations, VOC-202012/01 (also called VOC-20DEC-01 or the B.1.1.7 variant and labelled as Alpha by WHO (World Health Organization)), was first detected in the United Kingdom (UK) on December 14, 2020, with the total number of confirmed cases amounting to 249,637 [3,4], but was then reported by 93 countries on February 22, 2021. Thus far, 1,080,158 cases have been reported in 181 countries, and the top three countries most adversely affected by this variant are the UK (260,644 cases), the United States of America (USA) (216,352 cases), and Germany (101,785 cases) [5]. The VOC-202012/01 variant was followed by other mutations, including the B.1.351 variant (WHO label: Beta) which broke out in South Africa in September 2020, the P.1 variant (WHO label: Gamma) in Japan and Brazil in December 2020, the B.1.617.2 variant (WHO label: Delta) in India in December 2020, and the AY.1 variant (WHO label: Delta plus) in India in April 2021 [6].

Although the accuracy of the tests required to establish a definitive diagnosis of SARS-CoV-2 has already come under dispute, the prevalence of performing routine laboratory tests for diagnosis and treatment is still considerably high [7]. Being highly sensitive, specific, and reliable, Real-Time Polymerase Chain Reaction (RT-PCR)-based methods also suffer a range of disadvantages, such as requiring sophisticated laboratories and trained technicians, using a complex test algorithm, and having a lengthy turnaround time [8]. Though cost-effective PCR methods have been suggested, their cost to health care is undeniably massive, considering the efforts made to identify mutation types.

Within this context, the present study aims to study possible differences between individuals testing positive for SARS-CoV-2 with and without the VOC by using less costly complete blood count analytes and quickly analyzing the samples and ratios derived from these analytes. For this purpose, we assessed if there were statistical differences between neutrophil, lymphocyte, platelet, and Red Blood Cell Distribution Width-Standard Deviation (RDW-SD) levels among CBC parameters, as well as the neutrophil-lymphocyte ratio (NLR) and platelet-lymphocyte ratio (PLR) between those with vs without VOC in COVID-19 positive patients.

## Materials and methods

Study design

This study received ethics approval from the Ethics Committee for Clinical Investigations of Pamukkale University Training and Research Hospital, Denizli, Turkey, numbered 2021/09, dated April 27, 2021. A retrospective cross-sectional study was performed over the course of two months (from May to June 2021) on 212 patients with COVID-19 positive who presented to the emergency department (ED) of a tertiary hospital with COVID-19 symptoms and took the SARS-CoV-2 PCR and CBC tests, respectively. The laboratory test results and demographic information were retrieved from the public health management system retrospectively. The details of the PCR-confirmed SARS-CoV-2 positive patients and their hospitalization data were also gathered from the system. In addition, their VOC-202012/01 mutation status was confirmed by the public health management system.

Combined naso-oropharyngeal swabs collected from the eligible patients were analyzed with Real-Time Polymerase Chain Reaction** **(RT-PCR) tests. The CBC analysis was performed on the Mindray BC-6800 system using the electrical impedance and optic density method (Mindray Corp. Ltd., Shenzhen, China). Neutrophil/lymphocyte and platelet/lymphocyte ratios were calculated in line with the CBC results. In CBC, which contains neutrophil, lymphocyte, and platelet counts, we analyzed the parameters that we used for computing the ratios.

Statistical analysis

Two models were developed in accordance with the ratio and hematological analytes derived from the laboratory data. The data were analyzed with the Statistical Package for the Social Sciences (SPSS) v25.0 package program (IBM Corp., Armonk, USA). Continuous variables were presented as mean ± standard deviation, median (IQR: 25th-75th percentiles), and categorical variables were provided as numbers and percentages. The Kolmogorov-Smirnov test was used to investigate whether the variables met or violated the parametric test assumptions. The selection of appropriate tests for inferential statistics was made based on these normality assumptions. The Mann-Whitney U test and Kruskal-Wallis analysis of variance (post hoc: Bonferroni) were performed for the comparison of independent group differences. A logistic regression analysis was conducted to identify the risk factors. The receiver operating characteristic (ROC) analysis method was used to assess the performance of the given values. As a result of the ROC analysis, the Youden Index value was used to specify the most appropriate cut-off point. Following the analyses with the optimal cut-off point derived from Youden Index values, the area-under-the-curve (AUC) values ​​were obtained, and the performance results were analyzed. The statistical significance was set at p<0.05 for all the analyses.

## Results

Of the 212 individuals presenting to the ED between May and June 2021 and testing positive for SARS-CoV-2, 113 were male and 99 were female. Their mean age was 50.71±19.56 (min-max: 18-90). While no mutation was detected in 110 (51.9%) patients, the VOC-202012/01 mutation was observed in 102 patients (48.1%). Of 136 in-patients, 43 were hospitalized in the infectious diseases (ID) service, 67 in the chest diseases (CD) service, and 26 in the anesthesia intensive care unit (ICU). Note that infectious disease and chest disease services are the sub-disciplines of internal medicine under the Turkish healthcare system.

The age, as well as C-reactive protein (CRP), lactate dehydrogenase (LDH), neutrophil-lymphocyte ratio (NLR), and platelet-lymphocyte ratio (PLR) levels, were significantly higher in patients with mutation than without (p<0.0001). On the contrary, hemoglobin (HGB), hematocrit (HCT), eosinophil, and lymphocyte levels were significantly elevated in the patients without mutation (p<0.0001). Additionally, the mean age was 44.16 ± 18.56 years in those without mutation, whereas it turned out to be 57.76±18.19 years in the group with mutation (p<0.0001). In terms of hospitalization, 76 (35.8%) patients were followed up at home, while 43 (20.3%) were hospitalized in the ID service, 67 (31.6%) in the CD service, and 26 (12.3%) in the anesthesia ICU. Table 1 details the comparison of the patients’ laboratory data with the services in which they were hospitalized. Another aspect deserving attention is that the NLR and PLR values yielded a significant difference between the non-hospitalized population and those admitted to the anesthesia ICU (p< 0.001).

**Table 1:** Comparison of laboratory data with the services where the patients were hospitalized CRP: C-reactive protein, LDH: Lactate dehydrogenase, HGB: Hemoglobin, HCT: Hematocrit, RDW-SD: RDW-Standard deviation, NLR: Neutrophil-lymphocyte ratio, PLR: Platelet-lymphocyte ratio

	No hospitalization (n=76)	Infection Diseases Service (n=43)	Chest Diseases Service (n=67)	Anesthesia Intensive Care Unit (n=26)	p-value
Age	42.42 ± 17.56	43.44 ± 18.97	59.01 ± 17.28	65.54 ± 14.09	0.0001* bcde
	39 (28 - 54.75)	39 (27 - 61)	61 (47 - 72)	63.5 (56 - 77.25)	
CRP	22.23 ± 46.56	30.78 ± 51.12	78.9 ± 82.28	111.17 ± 74.5	0.0001* bcde
	2.81 (0.76 - 16.55)	5.63 (1 - 57.85)	51.47 (15.61 - 125.45)	103.84 (63.31 - 138.36)	
LDH	210.12 ± 84.46	239.64 ± 82.94	306.61 ± 160.52	400.56 ± 188.1	0.0001* bce
	186 (156 - 215.5)	208 (188.5 - 291)	269 (196.25 - 407.25)	359 (247.5 - 547)	
HGB	13.49 ± 2.13	14.34 ± 1.6	12.61 ± 2.39	11.32 ± 2.2	0.0001* cde
	13.55 (11.93 - 15.1)	14.5 (13.4 - 15.4)	13.2 (10.8 - 14.4)	11.05 (9.95 - 13.65)	
HCT	40.14 ± 5.54	42.02 ± 5.12	38.01 ± 6.7	33.91 ± 5.76	0.0001* cefd
	40.35 (36.8 - 43.88)	42.8 (39.1 - 45.3)	39.8 (33.4 - 42.8)	33.25 (29.63 - 39.7)	
Eosinophil	0.11 ± 0.13	0.14 ± 0.16	0.05 ± 0.07	0.04 ± 0.07	0.0001* bcde
	0.07 (0.02 - 0.17)	0.08 (0.02 - 0.23)	0.02 (0 - 0.07)	0.01 (0 - 0.04)	
Neutrophil	4.97 ± 2.69	7.49 ± 11.21	5.71 ± 3.64	5.68 ± 3.19	0.648
	4.52 (3.51 - 5.53)	4.65 (3.45 - 7.5)	4.57 (2.85 - 7.85)	5.22 (3.77 - 7.59)	
Lymphocyte	2.08 ± 2.13	1.89 ± 0.89	1.46 ± 1.03	0.79 ± 0.5	0.0001* bcdef
	1.88 (1.28 - 2.43)	1.85 (1.17 - 2.26)	1.25 (0.86 - 1.81)	0.72 (0.44 - 0.94)	
Platelet	256.95 ± 94.26	229.16 ± 62.37	214.45 ± 103.05	196.08 ± 105.83	0.003* bc
	261 (202.5 - 292.75)	224 (190 - 282)	213 (149 - 265)	156 (119.75 - 272.5)	
RDW-SD	41.69 ± 5.96	40.29 ± 2.41	42.8 ± 6.78	44.91 ± 8.89	0.093
	40.45 (38.35 - 43)	39.7 (38.4 - 41.7)	41.3 (38.6 - 43.9)	42.2 (39.53 - 46.85)	
NLR	3.43 ± 3.51	5.61 ± 10.85	5.42 ± 4.83	9.84 ± 7.91	0.0001* bcef
	2.27 (1.65 - 3.51)	2.74 (1.57 - 4.54)	3.48 (2.34 - 7.15)	7.67 (4.12 - 13.53)	
PLR	157.56 ± 86.91	143.12 ± 68.65	193.53 ± 131.59	332.88 ± 333.39	0.0001* ce
	129.04 (102.4 - 180.83)	123.16 (96.46 - 175.28)	170.53 (100 - 264.71)	241.01 (150.38 - 349.71)	
	0.2 (-1.02 - 1.4)	0.02 (-0.9 - 0.89)	0.68 (-0.85 - 1.6)	1.84 (-0.31 - 3.86)	

**Figure 1:**
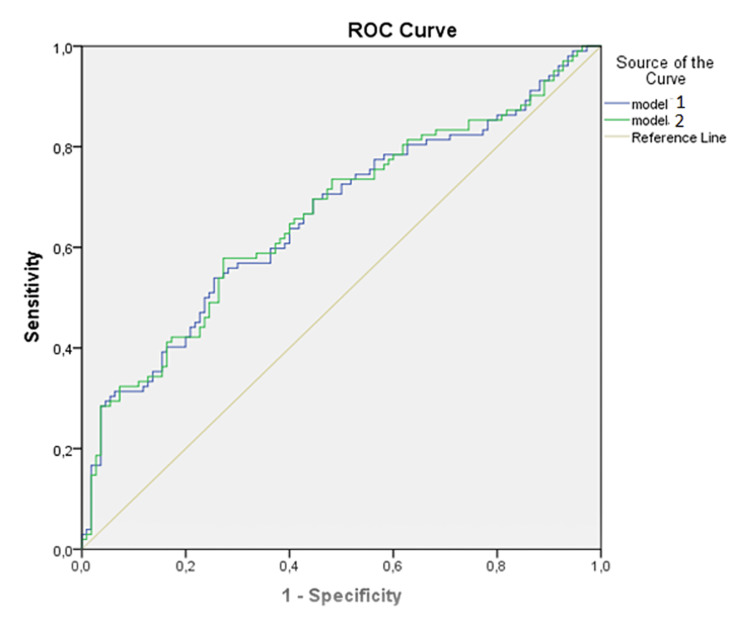
The receiver operating characteristic (ROC) curve of Model 1 and two regression models for discriminating between Sars-CoV2 mutation positive and negative groups. The receiver operating characteristic (ROC) curve of Model 1 and two regression models for discriminating between SARS-CoV2 mutation positive and negative groups. Model 1 and 2 regression models can be used for discriminating SARS-CoV2 and the mutation form

The ratio obtained from the laboratory data and the cut-off values of the models developed through the hematological analytes were derived from the ROC curves (Figure 1).

As far as the results from the univariate logistic regression models are concerned, CRP, LDH, RDW-SD, PLR, neutrophil-lymphocyte ratio-Red Blood Cell Distribution Width-Standard Deviation (NLR-RDW-SD) values as well as age and hospitalization to the CD service heightened the risk of mutation, while HGB, HCT, eosinophil, and lymphocyte values decreased the risk of the same. The multivariate models revealed that RDW-SD and PLR values were key indicators of the increased risk of mutation in the first model, while NLR values did not have a significant effect on the presence of mutation. In the second model, NLR values were excluded, and only the RDW-SD and PLR values were observed to heighten the risk of mutation (Table 2).

**Table 2:** Laboratory parameters used to predict the presence of mutation *p<0.05 statistically significant; O.R: Odds Ratio; C.I: Confidence Interval; ID: infectious diseases, CD: chest diseases, AICU: anesthesia intensive care unit; CRP: C-reactive protein, LDH: Lactate dehydrogenase, HGB: Hemoglobin, HCT: Hematocrit, RDW-SD: RDW-Standard deviation, NLR: Neutrophil-lymphocyte ratio, PLR: Platelet-lymphocyte ratio

		p-value	O.R.	95% C.I.for O.R.	
				Lower	Upper
Univariate	Age	0.0001*	1.039	1.023	1.056
	Gender	0.354	1.292	0.752	2.219
	CRP	0.0001*	1.01	1.005	1.015
	LDH	0.0001*	1.006	1.003	1.009
	HGB	0.0001*	0.682	0.59	0.788
	HCT	0.0001*	0.879	0.835	0.926
	Eosinophil	0.001*	0.007	0	0.125
	Neutrophil	0.457	0.98	0.928	1.034
	Lymphocyte	0.028*	0.701	0.51	0.962
	Platelet	0.599	0.999	0.996	1.002
	RDW-SD	0.001*	1.092	1.036	1.151
	NLR	0.243	1.027	0.982	1.074
	PLR	0.005*	1.004	1.001	1.006
	Hospitalization (ref: no hospitalization)	0.205	0.694	0.395	1.22
	Admission to ID (ref: no admission)	0.997	0	0	0
	Admission to CD (ref: no admission)	0.048*	2.006	1.005	4.003
	Admission to AICU (ref: no admission)	0.993	0.996	0.408	2.433
Multiple model 1	RDW-SD	0.004*	1.082	1.026	1.142
	PLR	0.022*	1.003	1.001	1.006
	NLR	0.65	0.988	0.936	1.042
Multiple model 2	RDW-SD	0.004*	1.083	1.026	1.142
	PLR	0.016*	1.003	1.001	1.006

## Discussion

Based on our final analysis, RDW-SD, RDW, NLR, and PLR indexes as well as CRP and LDH values remained higher in the patients with VOC-202012/01 mutation (p<0.0001) than in those without mutation, while hemoglobin and hematocrit counts and ratios, as well as eosinophil and lymphocyte counts, were lower in the patients with mutation (p<0.0001).

A substantial body of laboratory research has addressed the COVID-19 disease since its declaration as a worldwide pandemic. Although these studies suffer many limitations, they have nevertheless yielded insightful implications for establishing diagnosis and monitoring prognosis.

Seyit et al. compared the 123 SARS-CoV-2 negative and 110 positive patients in terms of CBC and biochemical parameters, reporting that CRP, LDH, PLR, and NLR values were significantly elevated in the positive cohort (p<0.0001; p=0.038; p<0.0001; p=0.001, respectively). In contrast, eosinophil, lymphocyte, and platelet counts were lower in the positive patients (p<0.0001; p<0.0001; p<0.0001, respectively) [7]. In our study, RDW-SD, Red Blood Cell Distribution Width (RDW), NLR, and PLR indexes in addition to CRP, LDH, NLR, and PLR values were high in the patients with the VOC-202012/01 mutation, while the hemoglobin and hematocrit counts and ratio, as well as eosinophil and lymphocyte counts, remained low. We did not note a significant difference in the neutrophil and platelet counts between the cohorts with and without the VOC-202012/01 mutation. We also observed that the VOC-202012/01 mutation further increased the CRP, LDH, NLR, and PLR levels while reducing the number of eosinophils and lymphocytes proportionally. Our findings revealed that the aforementioned parameters coupled with the models created with RDW-SD, which have drawn an increasing amount of attention recently, are statistically significant indicators for distinguishing the mutation. Kaeley et al. analyzed 350 COVID-19 positive patients, reporting that the patients with saturation below 94 had higher absolute neutrophil and NLR than those above 94. These results seem to confirm our findings that NLR was significantly higher in patients with the mutation, and this suggests that VOC-202012/01 mutation tends to reduce oxygen saturation [9].

RDW is a numerical measure of the size variability of circulating erythrocytes. Elevated RDW levels imply that the size distribution of red blood cells yields a greater variation than normal RDW values and signal bone marrow dysfunction induced by vitamin B12, folate, or iron deficiencies [10]. Pouladzadeh et al. divided 331 patients with COVID-19 into ‘severe’ and ‘non-severe’ cohorts and evaluated their RDW-SD levels [11]. They reported that the threshold RDW-SD level between 43 and 47 fl indicated high specificity (90.1-91.4%) for diagnosing non-severe illness and no risk of mortality, while RDW-SD>47 is indicative of severe symptoms and a high risk of mortality. With regard to our study, the significant difference in RDW-SD between the cohorts with and without the VOC-202012/01 mutation (p=0.007) suggests that more bone marrow dysfunction is evident among the patients with the mutation. Although RDW-SD did not significantly influence hospitalization (p=0.093), the highest RDW-SD values were noted in patients admitted to the anesthesia ICU. Among the SARS-CoV-2-positive patients, a significant difference was revealed between the patients with the VOC-202012/01 mutation and those without the mutation (p=0.007).

Wang et al. analyzed the hematological data and indexes of 45 patients who developed acute respiratory syndrome induced by moderate and severe SARS-CoV-2 [12]. They concluded that the combined index of NLR + RDW-SD proved to be a valuable diagnostic tool in predicting the clinical severity of the COVID-19 disease and following up with these patients. Corroborating these findings, our study suggests that the prediction models developed by combining RDW-SD with NLR and PLR were significantly higher in patients with the VOC-202012/01 mutation (p=0.001). Furthermore, the PLR values were significantly different between the non-hospitalized patients and their ICU counterparts, and between the patients in the ID service and their ICU counterparts (p=0.0001). Our dataset has provided the clinical evidence that NLR and PLR values elevate significantly when patients are admitted to the anesthesia ICU.

Pujani et al. carried out a two-month cross-sectional study in which 506 patients with COVID-19 and 200 healthy controls were monitored [13]. In this study, leukocytosis, neutrophilia, lymphopenia, and monocytosis were identified as characteristic indicators of COVID-19 cases, while the NLR and NLR+RDW-CV (red blood cell distribution width-coefficient of variation) index turned out to be the most effective CBC parameters in distinguishing mild, moderate, and severe cases respectively. Besides, the researchers evaluated the diagnostic value of each index by comparing it to the AUC area in ROC curves, concluding that the indicator with the highest AUC area was the combined NLR and RDW-SD index (0.871, p=0.000). Our study also revealed that the NLR and RDW-SD index and the increase in PLR were significantly different in laboratory and clinical terms. Two previous reports addressed this indicator in the diagnosis and follow-up of the patients with COVID-19 [12,13], but our study documents the first clinical evidence of the increase in the combined NLR, RDW-SD, and PLR index for individuals with mutations.

In their trial on patients with and without cardiovascular disease who suffered from SARS-CoV-2-related pneumonia, Petelina et al. prospectively studied the blood samples of both cohorts during their hospitalization as well as three months after their discharge [14]. They noted a significant elevation in hematocrit (HCT), mean corpuscular volume (MCV), and MCH (mean corpuscular hemoglobin) in both patient cohorts, and a decrease in mean corpuscular hemoglobin concentration (MCHC) and RDW-CV in both cohorts (p<0.001 for all parameters). They also identified a reduction in erythrocyte sedimentation rate (ESR) levels in the cohort with cardiovascular diseases. However, our results are indicative of a reverse situation, documenting reduced hemoglobin and HTC levels and increased NLR, PLR, and NLR RDW-SD values in the patients with the VOC-202012/01 mutation.

In their meta-analysis study, Simadibrata et al. reviewed 38 articles to explore whether NLR levels play a promising prognostic role in the severity of symptoms and mortality of COVID-19 patients [15]. They studied 5699 patients with severe clinical manifestations and 6033 non-survivors of COVID-19 patients. They associated high NLR levels on admission with severe progression of the disease and high risk of mortality, recommending that the patients with high NLR levels should be prioritized in the early risk stratification. Considering the difference in NLR values ​​on admission, the difference in NLR between the surviving and non-surviving patients was reported to be greater than the difference between clinically severe and non-severe patients. In addition, those with increased NLR levels on admission showed roughly twice the risk of death when compared to those with normal NLR levels. Of 38 studies reviewed, four studies reported that NLR levels between 3.3 and 5.9 can be set as the optimal threshold value for predicting clinical severity, while two studies establish the range of 7.9-11.8 to be an indicator of mortality. In our study, the NLR value for the patients with and without VOC-202012/01 mutation was calculated as 5.87±5.32 and 4.74±7.93 respectively, which confirms the published reports suggesting that NLR can be set as a parameter for the clinical aggravation of patients afflicted with VOC-202012/01 mutation (Table 1). Considering the significant role of NLR levels in the hospitalization status of our patients who tested positive for COVID-19, we identified a significant difference between non-hospitalization (mean±SD; 3.43±3.51) and admission to ID service (mean±SD; 5.61±10.85), non-hospitalization (mean±SD; 3.43±3.51) and admission to anesthesia ICU (mean±SD; 9.84±7.91), admission to ID service (mean±SD; 5.61±10.85) and anesthesia ICU (mean±SD; 9.84±7.91), and admission to CD service (mean±SD; 5.42±4.83) and anesthesia ICU (mean±SD; 9.84±7.91) (Table 2). Among these four groups of patients with COVID-19, the NLR values remained the lowest in the home follow-up group, while they were markedly elevated for the in-patients admitted to the CD and ID services. We identified the highest NLR values in the patients admitted to the anesthesia ICU. All this information implies that an elevation in NLR values may be associated with an increase in the clinical severity of COVID-19 in patients.

Limitations

The results reported by our study might have been affected by some limitations. First, our study sample consists of a limited number of patients admitted to a single hospital and service (only the ED). Considering the patients afflicted with variants of SARS-CoV-2, new mutations may emerge over time, and some changes could occur in the mutation-induced parameters, thereby altering the ways and tools for risk assessment. In addition, our results are based on retrospective data. Some of the blood parameters were included within the scope of our study, thus it might be more appropriate to assess them in combination with other laboratory findings. This study also did not take into consideration the treatment process, tomography imaging, and discharge status of the patients. Finally, we did not work with a scoring system for checking the health status of the patients in their follow-up.

## Conclusions

Our study suggests that the hematological analytes, the ratios obtained from these analytes, and the models created through these ratios in patients presenting to the ED with COVID-19-like symptoms and having positive RT-PCR test results were significantly different in those with and without the VOC-202012/01 mutation. The primary disadvantages of relying on RT-PCR tests for the diagnosis of COVID-19 are the lengthy turnaround time, high false-negative rates, and high costs. On the other hand, hematological parameters, NLR and PLR ratios derived from these parameters, and models based on these ratios and RDW-SD are cheaper, more widely used, and can predict patients’ clinical conditions as well as their need for hospitalization or admission to ICU. The bottom line is that they can serve as reliable predictors in the assessment of patients coming down with the VOC-202012/01 mutation.
